# Around the Hospitals

**Published:** 1894-06-16

**Authors:** 


### Around the Hospitals.

[Notice to the Managers of Institutions.—Special space is now reserved for the insertion of notices of hospital meetings and festivals. The Editor requests that all notices may reach The Hospital Office, 428, Strand, at least one week before the date of each meeting.]

**University College Hospital.**—The year 1893 was a very busy one at University College Hospital, owing to the fact that during that period several other metropolitan hospitals were closed, and, consequently, part of their work devolved on this institution. Unfortunately, with the patients came no increase of funds to meet the expense of their treatment. Consequently, the very large debt of the hospital remains undiminished. The appeal, in lieu of a festival dinner, resulted in a collection of £1,083, of which, unfortunately, only £15 represented annual subscriptions. The hospital especially pleads for aid towards two objects in its scheme—the one being the permanent endowment of beds and cots, which has not occurred at all of late years; and the secondly, further contributions towards the building fund. New buildings are urgently needed, yet they cannot be commenced until the fund reaches £30,000, although a much larger sum will be required to complete the undertaking. Apart from the actual treatment in the wards is the Samaritan branch of the work at University College Hospital. This is a very important feature. There are five different funds in connection with this. The Yates Fund provides surgical appliances, dinners and beef-tea to out-patients, comforts to poor mothers, new milk for sick children, and money grants to poor patients on returning to their homes. The other four funds are devoted to the maintenance of patients at convalescent homes. The Wakley Fund has recently been devoted to the acquisition of two beds at a convalescent home for the use of patients. It will be seen, therefore, that within the scheme of this North London hospital poor patients have the fullest opportunity for recovery. These patients within the hospital numbered last year 3,228, and in the out-patient department 49,486. The cost per bed was £65 18s. 1d., and per occupied bed £73 6s. 11d.

**The Royal Sea Bathing Infirmary.**—This institution, situated at Margate, for the reception of scrofulous patients, is still suffering much for want of funds. Two-thirds of its beds are unoccupied, and consequently an immense amount of possible benefit is curtailed. It is true that subscriptions show an increase, as do also congregational collections, and interest was doubtless quickened by the interesting gathering on its behalf held by the Archbishop at Lambeth Palace. Better things, therefore, may be hoped for before very long. During the past year four hundred and fifty-five patients were treated in the hospital, out-patients numbering forty-eight. The total ordinary expenditure amounted to £7,018, whilst the income amounted to £7,265, showing that the policy at Margate is curtailment of work rather than debt.

